# Recurrent traumatic atlantoaxial rotatory subluxation: Case report

**DOI:** 10.1016/j.amsu.2020.04.005

**Published:** 2020-04-24

**Authors:** Singkat Dohar Apul Lumban Tobing, Irsan Abubakar, I Wayan Arya Mahendra Karda

**Affiliations:** a Department of Orthopaedics and Traumatology, Cipto Mangunkusumo General Hospital – Faculty of Medicine Universitas Indonesia, Jakarta, Indonesia; b Department of Orthopaedics and Traumatology, University of Syiah Kuala, Aceh, Indonesia

**Keywords:** Atlantoaxial rotatory subluxation, Gardner-wells tongs, Posterior approach, Surgical treatment, Transarticular screw

## Abstract

**Introduction:**

Atlantoaxial rotatory subluxation (AARS) is not uncommon in paediatric emergencies, however, the complications might be fatal. Long onset before presentation is correlated with higher recurrence and persistent deformity. There is no consensus on the treatment of AARS yet. Selected patients may benefit from conservative approaches; however, retention might be difficult, and subluxation may recur.

**Presentation of case:**

A 6-year-old boy was admitted to our institution with AARS for three months before admission. Typical Cock-Robin position was observed. Computed tomography (CT) indicated AARS Fielding and Hawkins grade III. We treated the case conservatively by closed reduction and cervical traction using Gardner-Wells tongs. However, poor compliance resulted recurrence of subluxation, so we decided to fuse the atlantoaxial joint using transarticular screws, posterior wiring, and autologous bone grafting. Posterior fusion resulted in a satisfactory outcome, in which the wound healed accordingly. Six months of follow up examination revealed normal motoric and sensory function. The patient was able to perform daily activities with no significant issues.

**Discussion:**

Patients with fixed deformity of more than three weeks have a higher rate for recurrence or persistent deformity, as reduction is harder and difficult to maintain. The use of posterior wiring alone is limited in maintaining reduction, while using transarticular screws alone is considered better in maintaining reduction; however, not providing it.

**Conclusion:**

The use of posterior cervical fusion using C-wire, transarticular screws, and autologous bone grafting may be applied in recurrent case of AARS to ensure adequate reduction and fixation of the atlantoaxial joint.

## Highlights

• AARS is not uncommon in the paediatric setting as the biomechanical property of the paediatric spine introduces more risk for dislocation.
• A 6-year-old boy was admitted with AARS for three months before admission. After treated conservatively, the subluxation reoccurred. We decided to fuse the atlantoaxial joint.
• Posterior cervical fusion using C-wire, transarticular screws, and autologous bone grafting may be applied in recurrent case of AARS to ensure adequate reduction and fixation of the atlantoaxial joint.

## 1 Introduction

Atlantoaxial rotary subluxation (AARS) is a rotation of the atlantoaxial complex that is maintained in a fixed position, resulting in either muscle spasm or mechanical failure to reduction [1]. AARS is not uncommon in the paediatric setting as the biomechanical property of the paediatric spine introduces more risk for dislocation [[1], [2], [3]]. One to four percent of all paediatric trauma admissions is due to cervical spine injuries with up to 60% of all cervical rotations occurring at the C1–C2 level [1]. Paediatric with AARS most commonly caused by trauma, which is often minor, or after head and neck surgery [3]. Other causes such as congenital abnormalities (e.g. Down's and Marfan's syndrome), infection (e.g. Grisel's syndrome, eosinophilic granuloma), neoplasm, and inﬂammation (rheumatoid arthritis) have all been described [4]. Treatment for AARS remains controversial with no established standard modalities. The only agreement is the duration of symptoms as the best guide for treatment [5]. Most AARS could be treated successfully by conservative therapy. However, for recurrent cases or with disruption of transverse ligament, maintenance of closed reduction is quite challenging and often unsuccessful, requiring initial posterior atlantoaxial fusion [6]. Here we present our experience managing a case of atlantoaxial rotary subluxation in a paediatric patient by closed reduction and cervical traction using Gardner-Wells tongs, which then followed by the application of C1–C2 posterior cervical spine fusion due to recurrence of AARS.

This report is complaint with consensus-based surgical case report guidelines, SCARE Guidelines [7].

## 2 Presentation of Case

**Fig. 1:**
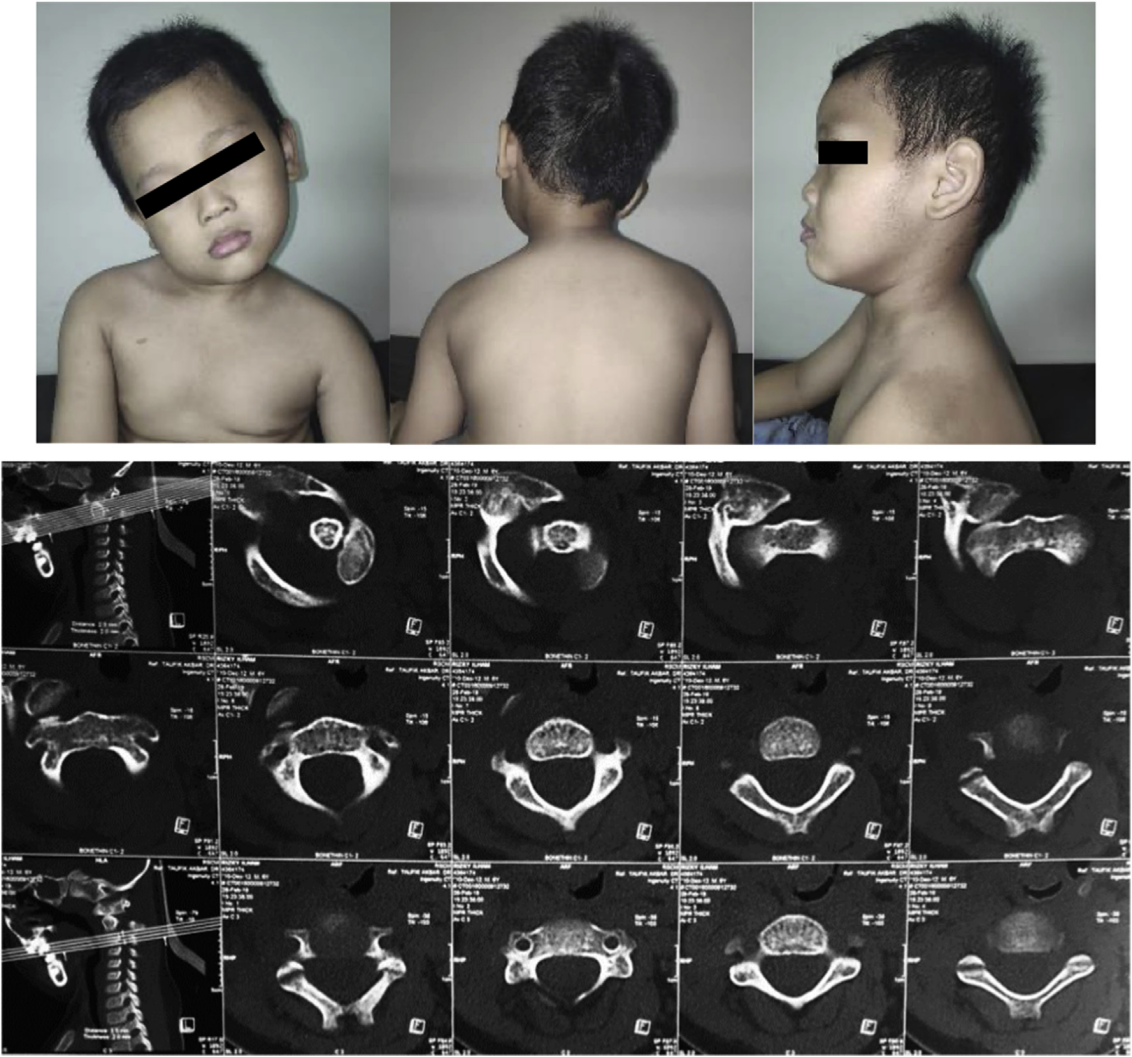
(a) The patient presented with the typical Cock-Robin.

A six-year-old boy was admitted to our institution with inability to move his neck for 3 months. Previously, the patient fell on his head and twisted his neck from a 2-m-height tree. There was no history of weakness on the extremities, and the patient did not have micturition and defecation complaints. The patient did not seek any medication. There was no known family history of the same condition or with joint laxity. The patient presented with a typical clinical sign of lateral flexion of the neck and contralateral rotation known as Cock-Robin head position (Fig. 1) with mild to moderate pain (visual analogue score of 3–4) during attempts of passive correction. Nuchal pain increased when an attempt was made to actively perform contralateral neck rotation. Marked sternocleidomastoid spasticity was observed along with decreased cervical spine range of motion. Neurological examination revealed normal sensory and motoric functions, without abnormalities in physiological reflex.

Computed tomography (CT) of the cervical spine revealed rotation and anterior dislocation of the atlas more than 5 mm (7 mm) indicating atlantoaxial rotary subluxation, Fielding, and Hawkins grade III. (Fig. 1). There was a bilateral anterior facet displacement with no fracture observed in the area.

**Fig. 2:**
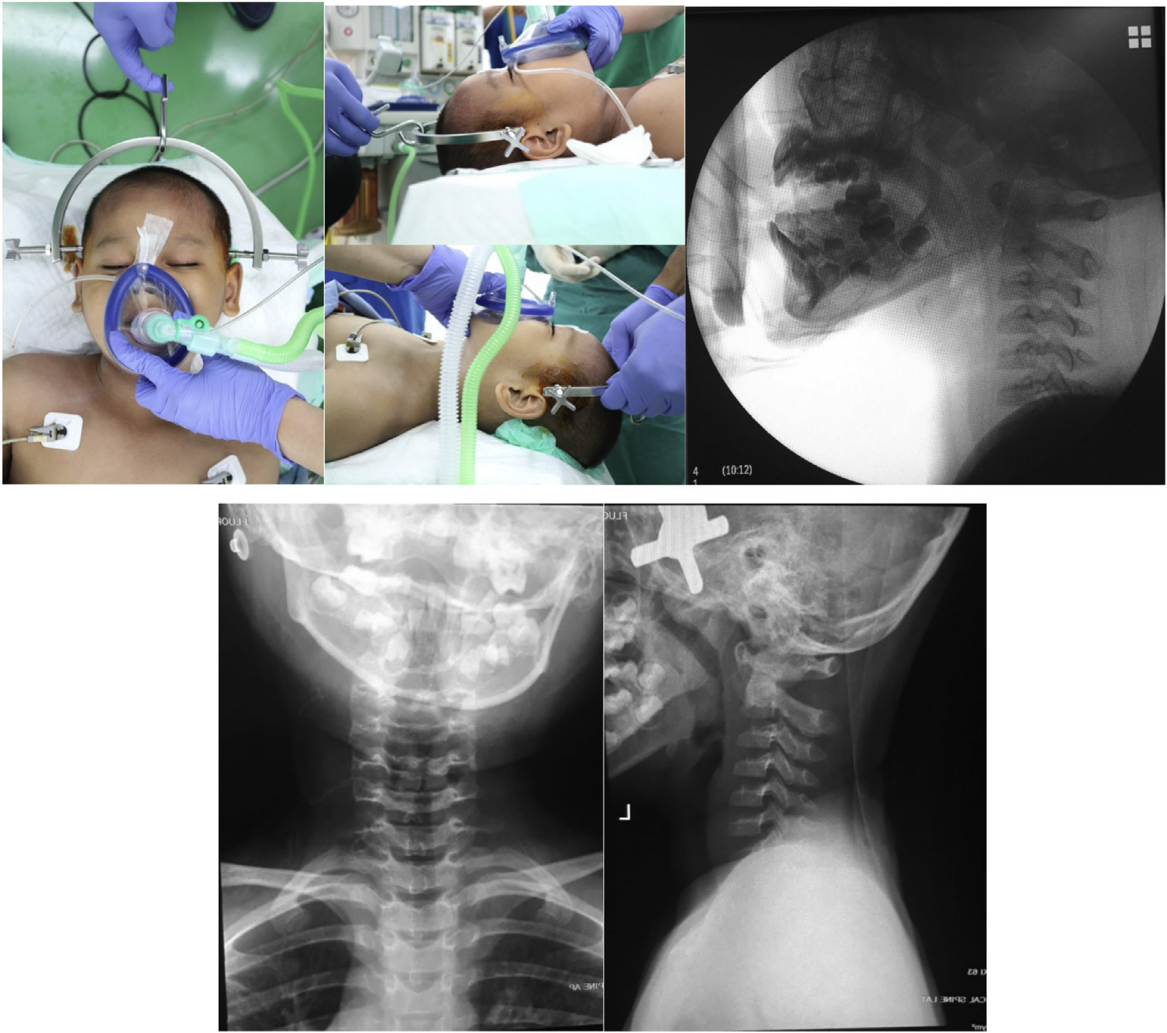
Closed reduction and cervical traction using Gardner-W.

**Fig. 3:**
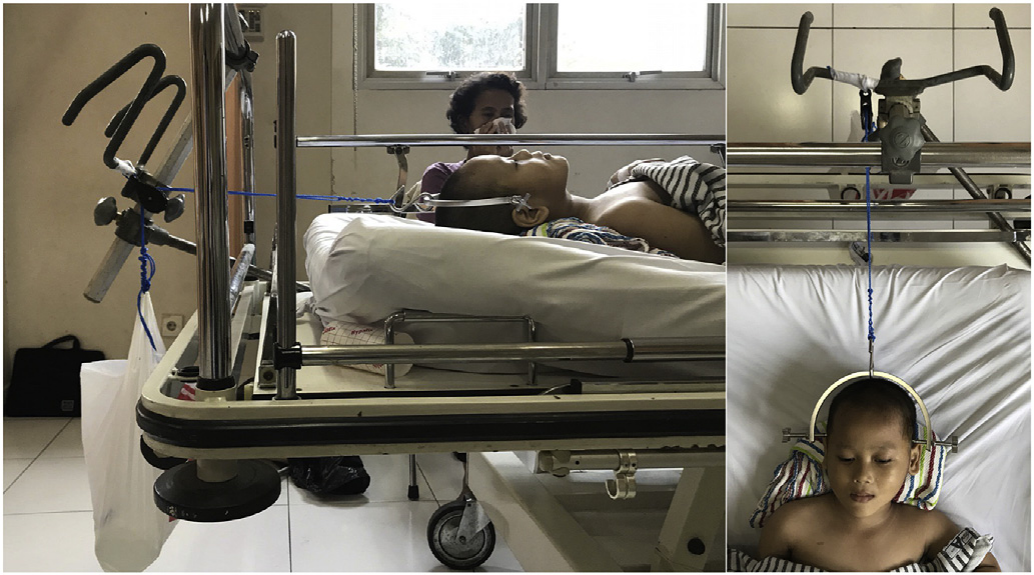
GWT was maintained with gradual increasing of weight f.

Closed reduction under general anaesthesia and cervical traction using Gardner-Wells tongs were performed for this patient. The procedure was performed by our senior Orthopaedic spine surgeon. The reduction was confirmed using intraoperative image intensifier and postoperative plain x-ray (Fig. 2). Traction was applied with gradual increase of weight reaching 3 kg. Cervical traction using GWT was maintained for 14 days until the patient was discharged using Philadelphia neck collar (Fig. 3). No neurovascular impairment was found during the hospital stay.

**Fig. 4:**
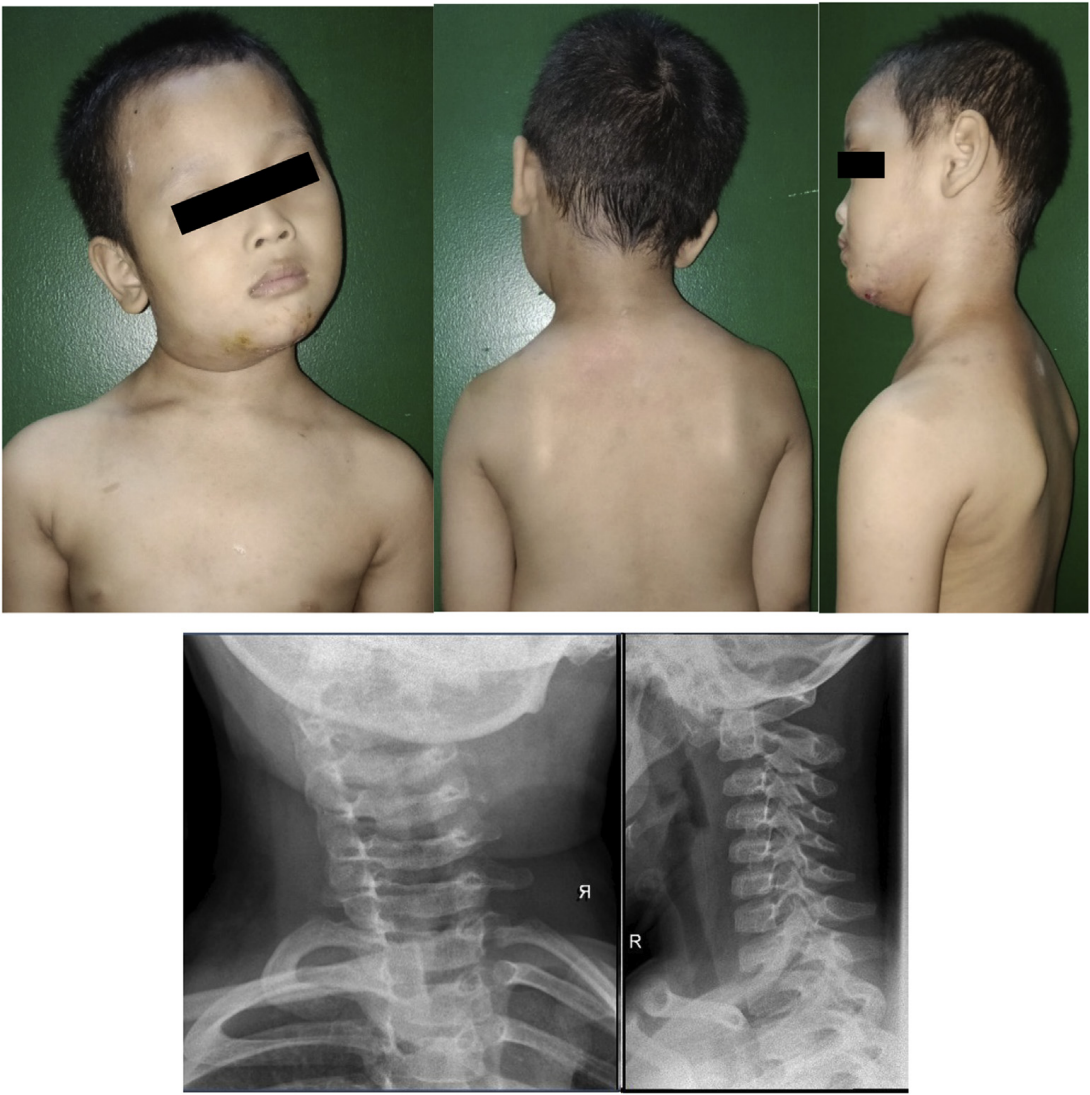
Recurrent AARS occurred 1 month after the initial cons.

Subluxation was reduced successfully for another two weeks, however, it failed and the AARS reoccurred. The patient got his neck twisted again while changing position during sleep. The patient didn't use brace for most times. The patient presented with another typical cock-robin position and tenderness VAS 3–4 during attempted passive correction. There was also spasticity of the sternocleidomastoid and decreased of cervical spine range of motion. No disturbance in the neurological aspect of the upper and lower limb. Cervical AP and Lateral X-ray was performed and showed recurrence of AARS (Fig. 4).

**Fig. 5:**
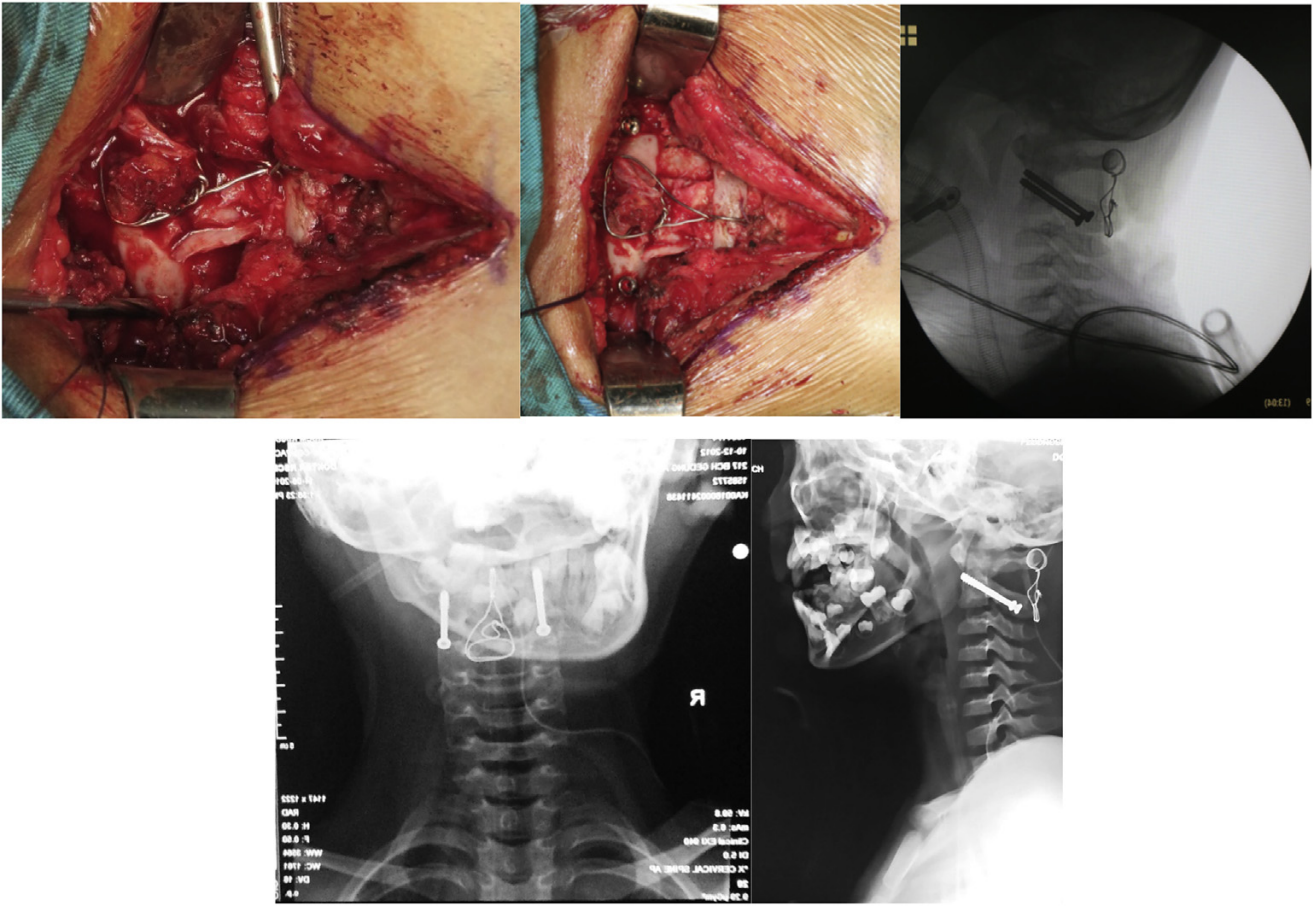
Wire and bone graft application, transarticular screw.

Definitive treatment was decided by performing fusion at the C1–C2 vertebrae. This procedure was performed by the same Orthopaedic surgeon who done the previous surgery. The patient was positioned prone during operation with the posterior approach opted for the procedure, then the posterior aspect of both atlas and axis were exposed. A 1.0 C-wire was inserted to the posterior part of the C1 and C2, with bone grafts harvested from the posterior iliac crest applied. Transarticular screwing was applied bilaterally using 3.5 half-threaded cannulated screw. (Fig. 5). The screw and C-wire placement was confirmed intraoperatively using fluoroscopy. The patient was directly admitted to the paediatric intensive care unit after the operation. The clinical and radiological follow-up indicated a complete clinical relief and satisfactory reduction, stable arthrodesis of atlantoaxial articulation. There was no neurological injury, wound infections, dural laceration, and suspected vertebral artery injury. The neck pain was lowered until VAS 1–2 post operatively. Hard collar neck was applied to be worn for four to six months, 24 hours daily, to prevent cervical flexion and extension. Post-operative compliance of the patient was good. The patient tolerated well using hard collar neck during his activities with no significant issues.

**Fig. 6:**
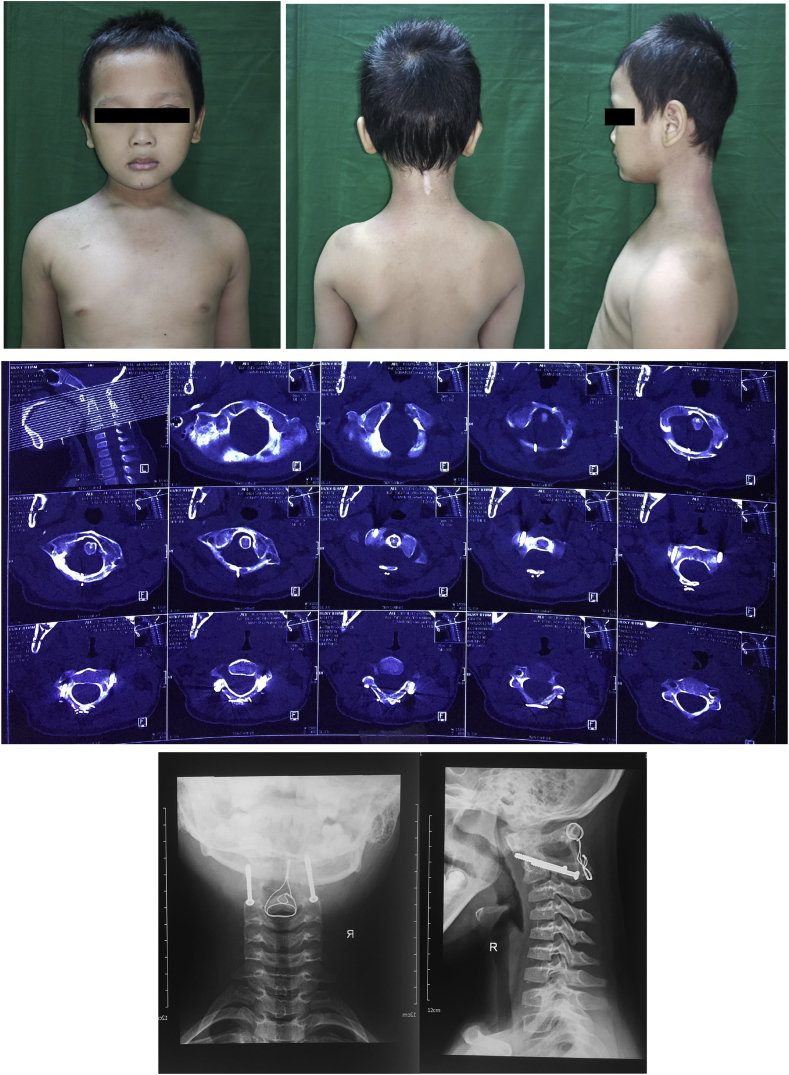
Clinical image, CT findings, and plain radiographs six.

At six months of follow up, the wound has healed accordingly and no complaint was found in the patient. There was no pain and tenderness complained by the patient that was measured using VAS score. Physical and neurological examination revealed the normal motoric function of +5 and normal sensory function of +2. The subluxated neck appeared straight per inspection and the Cock-Robin position disappears. The follow up CT findings showed reduction of atlantoaxial subluxation with C1 to anterior C2 dens length of 3.3 mm and good position of transarticular screws and wire (Fig. 6). The patient was able to perform daily activities with no significant issues.

## 3 Discussion

The ligaments of the paediatric spine and the craniovertebral junction are considerably laxer and the facet joints allow greater mobility [[1], [2], [3]]. AARS occurs more in children as the consequence of the relative weakness of ligaments and joint capsules, horizontal joint articulation, not fully developed supporting muscles and bigger head to body relationship [3,8,9]. The patients are usually present with a classic sign of torticollis: contralateral rotation and upper cervical spine inclination [[1], [2], [3],^10^]. Sudek et al. described there will be a palpable deviation of C2 spinous process in the same direction as head rotation in patients with AARS; ipsilateral sternocleidomastoid muscle spasm as an attempt to reduce the deformity; and the inability to counterrotate the head past midline [3]. This traumatic subluxation is mostly caused by disruption of the soft tissue components that keeps the atlantoaxial joint in place such as the transverse ligament [3,11].

Our patient presented with classical signs of AARS without neurological impairments. The onset of the subluxation was three months. No clear definition existed to determine the transition from acute to chronic AARS. Some experts suggested that acute AARS occurred below 8 weeks while chronic AARS was defined if the patient had symptoms for more than 3 months [1,12].

CT has been the modality of choice in cases of cervical trauma to assess bony injuries. However, the use of CT should be limited to high-risk injuries as this method of diagnosis poses patients to the high amount of radiation [13]. Establishment of diagnosis, in this case, is made by CT scanning with additional 3D reconstruction. This allows better visualization of the subluxation. On the other hand, the magnetic resonance imaging (MRI) is more beneficial to assess the soft tissue, e.g., spinal cord injury, ligament injury, etc. [13].

Fielding and Hawkins proposed a classification of AARS as assessed using the axial CT, divided into four types in general [1]. Special precaution should be made after establishing the diagnosis of type III and IV AARS, particularly [1]. Regarding the time of onset, there is no consensus on when to diagnose AARS as acute or chronic; however, the choice of treatment changes when patients present after one month of onset.

The main goal of AARS treatment is to restore/realign the cervical spine, achieved by performing immobilization and reduction of the subluxated joint. Options vary from conservative measures using medications, orthopaedic appliances, or even operative procedures. The duration of symptoms before admission may predict the treatment modality required and the possible outcome of the treatment. Orthopaedic appliances such as cervical halter traction can be used to reduce AARS by applying traction. Traction may assist the increase of spinal canal diameter, as well as lengthening and relaxing the surrounding muscles [2]. As the odontoid is distracted from the foramen magnum, reduction might occur [2].

After one month of successful closed reduction and cervical traction, our patient developed recurrent AARS. It should be taken into consideration that patients with fixed deformity of more than three weeks are had a higher rate for recurrence or persistent deformity, as reduction is harder and difficult to maintain [1,14]. Possible mechanisms for recurrence include over distension of the capsule joint, overstretching of stabilizing ligaments, and remodelling of the articulation of C1 and C2. It has shown that C2 facet and C1 lateral mass remodelling may begin to occur as early as 1 month after a chronic dislocation [5].

In cases where another subluxation occurs, it is recommended to reapply traction and the patient is immobilized for a longer period. If this approach fails the second time, usually surgical procedures follow [3].

For traumatic rotatory subluxation, Fielding and Hawkins reported 17 patients with an average delay of diagnosis of 11.6 months. Their treatment included skull traction followed by surgical arthrodesis, if needed. Surgical arthrodesis was performed on 13 of those patients, with 11 considered as good results. They found that recurrence of subluxation occurred in patients with C1–C2 ﬁxation of more than 3 months and these patients were best treated with fusion. This recommendation has since been supported by other studies [5].

Surgical procedures may be the treatment of choice in some cases to prevent further complications such as nerve decompression and respiratory failure that may ultimately lead to death. If disruption of the transverse ligament occurs, the best option would be to fuse the C1 and C2 by screw as it is associated with the smallest degree of range of motion limitation. Surgical procedures to reduce and fixate the C1 and C2, in this case, is by posterior method, which is C1–C2 transarticular screw fixation [2]. This approach alone can be used for reducible atlantoaxial dislocation, and other methods such as the anterior transoral decompression can be co-administered [15]. The use of posterior wiring alone is limited in maintaining reduction, while using transarticular screws alone is considered better in maintaining reduction; however, not providing it [5]. We performed transarticular screw fixation and posterior wiring, along with the addition of bone graft to ensure stable reduction and fixation of the atlantoaxial joint.

Six months after the procedure, the patient exhibited satisfactory results from the procedure, where the pain resides, and the wound recovered appropriately. Neurological status remains within normal limits with no new signs or symptoms arising.

## 4 Conclusion

The use of posterior cervical fusion using C-wire, transarticular screws, and autologous bone grafting may be applied in recurrent case of AARS to ensure adequate reduction and fixation of the atlantoaxial joint.

## Ethical approval

This is a case report; therefore, it did not require ethical approval from ethics committee. However, we have got permission from the parents to publish his data.

## Consent

We have written and signed informed consent obtained from the parents to publish this case report and accompanying images.

## Provenance and peer review

Not commissioned, externally peer reviewed.

## Declaration of competing interest

The authors declare that there is no conflict of interest regarding publication of this paper.

## Appendix A Supplementary data

Application Application
